# Role of Platelet-Rich Plasma (PRP) in the Management of Stage III and IV Degenerative Disc Disease

**DOI:** 10.7759/cureus.79504

**Published:** 2025-02-23

**Authors:** Orestis V Paliaroutas, Dimitrios Stergios Evangelopoulos, Elias Vasiliadis, Nikoleta Stanitsa, Georgios Zouris, John Vlamis

**Affiliations:** 1 First Orthopedic Department, National and Kapodistrian University of Athens School of Medicine, KAT Hospital, Athens, GRC; 2 Third Orthopedic Department, National and Kapodistrian University of Athens School of Medicine, KAT Hospital, Athens, GRC; 3 Cardiothoracic Surgery Department, Evangelismos Hospital, Athens, GRC; 4 Fifth Orthopedic Department, Asklepieion Voulas General Hospital, Athens, GRC

**Keywords:** degenerative disc disease, discogenic low back pain, disk degeneration, platelet-rich plasma, prp

## Abstract

Degenerative disc disease is a common disorder that can significantly impact patients’ quality of life, leading to chronic pain and disability. Platelet-rich plasma (PRP) therapy is emerging as a potential treatment for degenerative disc disease. The purpose of this review is to summarize the role of PRP in the management of degenerative disc disease types III and IV.

This is a scoping literature review. The online database PUBMED was used, and papers were searched using the keywords: ("PRP" OR "platelet-rich plasma") AND ("degenerative disk disease" OR "disk degeneration" OR "intradiscal injection" OR "discogenic pain" OR "intervertebral disc degeneration" OR "degenerative disk disease" OR "intervertebral disc disease"). Clinical studies evaluating the role of PRP in the management of stage III and IV degenerative disc disease were included in the study. Systematic reviews, animal studies, in vitro studies, case reports, study designs, case reports, and studies in languages other than English were excluded.

The present study includes 14 studies. PRP has been found to promote tissue regeneration and modulate inflammatory response in degenerated discs. PRP can be administered mostly intradiscally but also epidurally. The benefits of PRP use include pain reduction, improvement of functionality, and low risk of adverse events. The effect of intradiscal PRP injections is similar to steroid injections. A higher concentration of platelets is associated with enhanced clinical outcomes.

PRP therapy represents a promising avenue for tissue healing and regeneration across degenerative disc disease. While the evidence supporting its efficacy is encouraging, further research is needed to elucidate its mechanisms of action, optimize treatment protocols, and expand its clinical applications.

## Introduction and background

Platelet-rich plasma (PRP) therapy has emerged as a promising treatment modality for a wide range of medical conditions, owing to its regenerative properties and potential to enhance tissue healing. PRP is prepared by centrifuging whole blood to separate the platelet-rich fraction from other blood components. The resulting PRP contains a concentrated mixture of platelets, growth factors (such as platelet-derived growth factor (PDGF), transforming growth factor-beta (TGF-β), and vascular endothelial growth factor (VEGF)), cytokines, and other bioactive molecules that work synergistically to promote tissue repair, angiogenesis, and cell proliferation. Upon activation at the site of injury or tissue damage, platelets release their granules, which contain a plethora of bioactive molecules. These growth factors and cytokines stimulate various cellular processes, including cell migration, proliferation, differentiation, and extracellular matrix synthesis. By enhancing the recruitment and activity of reparative cells, PRP accelerates the healing process and facilitates tissue regeneration [1-3].

PRP therapy has been utilized in a wide range of medical specialties and clinical scenarios, including the treatment of musculoskeletal injuries and osteoarthritis, athletic injuries, wound healing and hair restoration, and aesthetic medicine. Its use is generally considered safe, as it utilizes autologous blood components and minimizes the risk of immunogenic reactions or disease transmission. However, adverse events such as pain at the injection site, local inflammation, and transient exacerbation of symptoms have been reported in some patients [4-7].

Degenerative disc disease is a condition characterized by structural changes and functional impairment of the intervertebral discs in the spine. While it is commonly associated with aging, it can also occur as a result of injury, repetitive stress, or genetic predisposition [8-10].

Intervertebral discs are avascular fibrocartilaginous structures located between adjacent vertebrae interposed with a cartilaginous endplate. They consist of a gel-like nucleus pulposus surrounded by a tough, fibrous outer layer called the annulus fibrosus. The outer annulus fibrosus consists of collagen I-rich fibrocartilage lamellae, while the inner nucleus pulposus is composed of a proteoglycan-rich gel-like matrix with less organized type II collagen fibers. The endplates prevent collision between the intervertebral discs while also providing nutrients and oxygen to the intervertebral discs through passive diffusion. The intervertebral discs act as shock absorbers, providing structural stability and flexibility to the spine [11].

Degenerative disc disease involves a multifactorial process characterized by progressive changes in the structure and function of intervertebral discs. These changes occur progressively with aging, may have a genetic predisposition, and can be exacerbated by disease and/or injury. They may include loss of disc height, dehydration, tears or fissures in the annulus fibrosus, and migration of nucleus pulposus contents into the annulus fibrosus. These contents contain high concentrations of pro-inflammatory cytokines that initiate chemical sensitization of the nociceptors found in the outer annulus fibrosus [12]. Other processes occurring during intravertebral disc degeneration include the loss of glycosaminoglycans, an increase in denatured type II collagen, and an increase in fibronectin fragments. Over time, these degenerative changes can lead to altered biomechanics, spinal instability, and compression of nearby nerves or spinal cord [13].

The most common symptom of degenerative disc disease is chronic lower back pain, which may be aggravated by activities that load the spine, such as bending, lifting, or sitting for prolonged periods. If the degenerative changes result in compression of spinal nerve roots, radicular symptoms may be present, such as pain, numbness, tingling, or weakness. Functional impairment, including limitations in mobility and activities of daily living, may also occur [14]. Diagnosis of degenerative disc disease is typically based on clinical evaluation, including a detailed history and physical examination. Imaging studies, such as X-rays, magnetic resonance imaging (MRI), and computed tomography (CT) scans, may be ordered to confirm the diagnosis and assess the extent of degenerative changes in the spine [14-16].

The Pfirrmann grading scale is a widely used classification system for assessing the degree of intervertebral disc degeneration based on MRI findings. It was developed by Pfirrmann et al. in 2001 and has since been adopted in clinical practice and research settings for evaluating the severity of disc degeneration. The scale assigns a numeric grade (ranging from I to V) to each intervertebral disc based on its appearance on T2-weighted MRI images (Table 1) [17].

**Table 1:** Pfirrmann grading scale for degenerative disc disease Reference: [17]

Stage	MRI findings	Characteristics
I	Normal disc structure with homogeneous hyperintense signal intensity of the nucleus pulposus and distinct distinction between nucleus pulposus and annulus fibrosus.	Well-hydrated nucleus pulposus, intact annulus fibrosus, and minimal to no evidence of degenerative changes.
II	Slightly decreased signal intensity of the nucleus pulposus, but still with clear distinction between nucleus pulposus and annulus fibrosus.	Mild loss of disc hydration, with minimal changes in disc height and integrity of the annulus fibrosus.
III	Intermediate signal intensity of the nucleus pulposus with unclear distinction between nucleus pulposus and annulus fibrosus.	Moderate loss of disc hydration, with decreased distinction between nucleus pulposus and annulus fibrosus, and possible signs of early disc degeneration such as annular tears or fissures.
IV	Hypointense signal intensity of the nucleus pulposus with a reduced or absent distinction between nucleus pulposus and annulus fibrosus.	Severe loss of disc hydration, with pronounced degenerative changes in the annulus fibrosus and nucleus pulposus, including disc bulging, herniation, and decreased disc height.
V	Severely hypointense signal intensity of the nucleus pulposus, with complete collapse of the disc space and loss of distinction between nucleus pulposus and annulus fibrosus.	End-stage degenerative changes, with complete loss of disc height, extensive disc collapse, and potential adjacent vertebral body changes such as osteophyte formation or endplate sclerosis.

Treatment of degenerative disc disease initiates with conservative measures, including physical therapy, non-steroidal anti-inflammatory drugs (NSAIDs), analgesics, and corticosteroid injections to alleviate pain and improve function [18]. In cases where conservative measures fail to provide relief or in the presence of severe symptoms, surgical interventions such as discectomy, laminectomy, spinal fusion, or artificial disc replacement may be considered [18-19].

Ongoing research efforts in degenerative disc disease focus on understanding the underlying mechanisms of disc degeneration, identifying novel therapeutic targets, and developing personalized treatment strategies based on patient-specific factors such as genetic predisposition, biomechanical factors, and molecular biomarkers. PRP therapy represents a promising, biologically based approach to treating degenerative disc disease. The purpose of this review is to summarize the role of PRP in the management of degenerative disc disease types III and IV.

## Review

Material and methods

This is a scoping literature review. The online database PUBMED was used, and papers were searched using the keywords: ("PRP" OR "platelet-rich plasma") AND ("degenerative disk disease" OR "disk degeneration" OR "intradiscal injection" OR "discogenic pain" OR "intervertebral disc degeneration" OR "degenerative disk disease" OR "intervertebral disc disease"). Clinical studies evaluating the role of PRP in the management of stage III and IV degenerative disc disease were included in the study. Systematic reviews, animal studies, in vitro studies, case reports, study designs, and studies in non-English language were excluded.

Results

A total of 101 studies were identified through the initial PubMed database search. After screening titles and abstracts, 21 studies were excluded as irrelevant. Full-text articles were assessed for eligibility (n = 80), of which 66 were excluded for the following reasons: review articles (n = 27), case reports (n = 5), research protocols (n = 1), animal studies (n = 17), in vitro studies (n = 11), and studies in languages other than English (n = 5). Ultimately, 14 studies were included in the qualitative synthesis.

Discussion

In recent years, PRP has emerged as a relatively non-invasive treatment option for degenerative disc disease after the failure of conservative management [20]. PRP has been found to act by providing a high concentration of cytokines, chemokines, and growth factors, including GF-β, insulin-like growth factor (IGF), PDGF, epidermal growth factor (EGF), fibroblast growth factor (FGF), and VEGF, which activate cell proliferation and differentiation of vascularized cells [21]. This potential for angiogenesis suggests that PRP administration may be useful in areas where vascularity is relatively preserved, including ligaments, tendons, and muscles [22-23]. On the other hand, the vascular supply to the intervertebral discs is quite limited; they rely on diffusion from adjacent vascularized tissues for nutrient delivery and waste removal [24-26]. So, one may suggest that PRP may have minimal effect on the degenerated discs. However, PRP has been found to have anti-inflammatory properties at the injected site mainly through the attenuation of the transactivation of the inflammatory regulator, nuclear factor kappa B (NF-κB), and by inhibiting the inflammatory enzymes cyclooxygenase-2 (COX-2) and cyclooxygenase-4 (COX-4), matrix metalloproteinases (MMPs), and disintegrins [24-26]. These anti-inflammatory properties of PRP make it a potential injectable option for the management of discogenic pain in degenerative disc disease.

The use of PRP in the treatment of degenerative disc disease includes certain benefits. As inflammation plays a significant role in the progression of degenerative disc disease and contributes to pain and tissue damage, the anti-inflammatory components of PRP may help reduce inflammation in the affected discs, thereby alleviating symptoms associated with degenerative disc disease. The growth factors contained in PRP have the potential to stimulate tissue repair and regeneration. By promoting the growth of new cells and extracellular matrix components, PRP may help restore the integrity and function of degenerated discs [27-29]. Moreover, PRP therapy may help alleviate pain associated with degenerative disc disease by reducing inflammation, promoting tissue healing, and improving disc hydration and function. Finally, PRP has been suggested to modulate the degenerative process in intervertebral discs by promoting anabolic processes and inhibiting catabolic processes. This modulation may decelerate the progression of degenerative disc disease and potentially improve the long-term outcomes for patients [30-32].

Technique

Peripheral venous blood is withdrawn, typically from the arm, using a standard blood collection technique. The collected blood is then undergoing centrifugation in a centrifuge machine, where the platelets are concentrated into the plasma, forming PRP. In some cases, an activator such as calcium chloride or thrombin may be added to the PRP to initiate the release of growth factors from the platelets [33-34].

With the patient in a prone position, the area where the injection will be administered is cleaned and sterilized, and local anesthesia is applied. Under fluoroscopic guidance, a thin needle (22G) is inserted into the center of the targeted intervertebral disc. The needle is advanced through the skin, muscles, and ligaments until it reaches the nucleus pulposus. The infiltration process is performed using an oblique angle of 25°-35° at the lateral margin of the superior articular process of the lower vertebra, between the inferior endplate of the upper vertebra and the superior endplate of the lower vertebra, and always lateral to the neuroforamen for nerve root preservation. Once the needle is correctly positioned, the PRP is injected into the nucleus pulposus of the intervertebral disc. After the injection, the patient may be monitored for a short period to ensure there are no immediate adverse reactions [33,35].

Clinical outcome

In a prospective trial by Levi et al., 22 patients (10 men, 12 women, median age: 47 years) with discogenic low back pain underwent intradiscal PRP injection. A 50% improvement in the pain intensity and a 30% decrease in the Oswestry Disability Index were observed in 14% of patients at one month, in 32% of patients at two months, and in 47% of patients at six months after injection [36].

In 2017, a preliminary clinical trial by Akeda et al. included 14 patients (8 men, 6 women, mean age: 34 years) with chronic low back pain and evidence of degeneration in one or more lumbar discs, undergoing intradiscal PRP injection. All patients were graded >3 in the Pfirrmann classification system. The mean platelet count of PRP was approximately four times greater than that of whole blood. After a mean 10-month follow-up, mean pain scores were significantly decreased at 1, 6, and 12 months. A more than 50% reduction of low back pain was observed in 71% of patients within one month after PRP-releasate injection. About 79% of patients showed a more than 50% reduction in physical disability scores one month after PRP-releasate injection. No significant radiographic changes were recorded. The mean T2 values did not significantly change after treatment [33].

In 2022, Akeda et al. published a long-term follow-up of the previous prospective clinical study. Eleven patients were left for long-term evaluation. Improvements in pain intensity and physical disability were sustained at an average of 5.9 years after the intradiscal PRP injection. In 91% of patients, more than a 30% improvement in pain intensity and physical disability was identified. A 14% decrease in disc height compared to baseline was recorded at 5.9 years [37].

A recent prospective observational study by Kirchner et al. included 32 patients (19 men, 13 women, mean age: 55 years) with cervical and lumbar chronic pain due to intervertebral disc degeneration (59 discs involved). Sixty-one percent of patients were classified as grades 3 and 4 in the Pfirrmann Grade. All patients received 2-3 series of intradiscal and epidural PRP injections under fluoroscopic guidance, with two weeks between each procedure. Patients were assessed at one, three, and six months after treatment. Pain intensity and disability showed a statistically significant reduction at one, three, and six months after injections (p < 0.001). Pain reduction of more than 30% was observed in 78% of patients one month after treatment and in 87% of patients six months after treatment [34].

Cheng et al. conducted a five-year to nine-year follow-up of a previous randomized controlled trial, evaluating the results of intradiscal PRP injections in a subset of patients. Nineteen patients (mean age: 41 years) with chronic low back pain and a grade 3-4 intervertebral disc degeneration received intradiscal PRP injections. The authors observed statistically significant improvements in pain and function (p < 0.001) at 5-9 years after PRP injections. Fifty-eight percent of patients were satisfied with the PRP injection [38].

A prospective trial by Zhang et al. included 31 patients (12 men, 19 women, mean age: 31 years) with refractory discogenic low back pain, who received a single intradiscal PRP injection. The results of the study showed that PRP injection was associated with a significant improvement in pain intensity and lumbar function [39].

In a multicenter, double-blind, randomized, placebo-controlled study, Zielinski et al. evaluated the effect of intradiscal PRP injection in patients with chronic discogenic pain. A total of 26 patients (12 men, 14 women) with a diagnosis of chronic lumbar discogenic pain, classified as grade >3 in the Pfirrmann classification system, were randomized to receive intradiscal PRP injection (n = 18) or saline (n = 8). The results of the study failed to demonstrate any significant superiority of PRP injections in terms of pain relief or functionality improvement [40].

Comparison to corticosteroids

A randomized, double-blind, active-controlled clinical trial by Akeda et al. was published in 2022. Sixteen patients (11 men, 5 women, mean age: 32 years) with discogenic low back pain were randomized to receive either PRP releasate intradiscal injection (n = 9) or corticosteroid (n = 7). All patients were classified as grade 4 in the Pfirrmann classification. In both treatment groups, pain intensity was significantly decreased, but the improvement was not statistically different between groups at two months. In comparison to the corticosteroid group, the PRP releasate group had a significantly improved disability score at 26 weeks and walking ability scores at one and two months. No significant differences in radiographic disc height and MRI grading score were noted. No important side effects were recorded [35].

A retrospective analysis of the previous randomized clinical trial of intradiscal injection of the releasate isolated from PRP by Akeda et al. in 15 patients with discogenic low back pain and MRI evidence of degenerative disc disease was published in 2023. All patients were classified as grade 4 in the Pfirrmann classification. Nine patients received PRP injections, and six patients received corticosteroid injections. No significant changes in radiographic parameters (segmental angulation or lumbar lordosis) or MRI phenotype were observed. Intradiscal injection of PRP releasate significantly improved low back pain and low back pain-related disability one year after the injection; however, the number of targeted discs and the presence of posterior high-intensity zones at baseline was negatively associated with treatment outcomes [41].

A recent comparative study by Jayasoorya et al. included 64 patients with lumbar intervertebral disc prolapse diagnosed and low back pain lasting at least one month. All patients were classified into two groups: group A (n = 32) underwent a single epidural injection with methylprednisolone, and group B (n = 32) underwent a single epidural PRP injection. In group B, pain intensity was decreased in comparison to group A after one hour and three months. The authors concluded that “PRP is an effective alternative to epidural steroid infiltration in managing chronic discogenic low back pain” [42].

Comparison to bone marrow concentrate

A multicenter, prospective, crossover, randomized, controlled trial by Navani et al. included 40 patients with degenerative disc disease and relevant MRI findings, randomized to receive either saline trigger point injection (n = 12), intradiscal PRP (n = 13), or bone marrow concentrate (n = 15). Both PRP and bone marrow concentrate were associated with statistically significant improvement in pain and function in comparison to placebo. No severe adverse events were recorded [43].

PRP and stromal vascular fraction

Stromal vascular fraction is a mixture of cells and various cell-derived components that are isolated from adipose tissue through a lipoaspirate procedure. It contains a variety of cell types, including mesenchymal stem cells (MSCs), adipose tissue-derived stem cells (ADSCs), endothelial cells, pericytes, and immune cells such as macrophages and lymphocytes, along with growth factors. Stromal vascular fraction is obtained through a process called enzymatic digestion and centrifugation of adipose tissue. Once isolated, stromal vascular fraction can be used for various purposes in regenerative medicine and tissue engineering due to its potential to differentiate into different cell types and its ability to promote tissue repair and regeneration. Stromal vascular fraction has been investigated for its therapeutic potential in treating conditions such as osteoarthritis, wound healing, and cardiovascular diseases, among others [44].

Comella et al. evaluated the safety and efficacy of administering stromal vascular fraction and PRP intradiscal to 15 patients with degenerative disc disease. Stromal vascular fraction was derived from a local tumescent liposuction that removed 60 ml of adipose tissue. The stromal vascular fraction was collected as a pellet, which was resuspended in 1-3 ml PRP. The stromal vascular fraction/PRP suspension was injected intradiscally. After one year of follow-up, no severe side effects were noted. Pain intensity, flexion, and quality of life were significantly improved. No significant impact was observed in scores of disability and depression [44].

Impact of platelet concentration

The efficacy of PRP therapy largely depends on the concentration of platelets in the plasma. A higher concentration of platelets means a higher concentration of growth factors and cytokines. This enhances the biological activity at the injury site, promoting faster and more effective tissue repair and regeneration. Higher levels of growth factors can attract more reparative cells to the injury site, improving the overall healing process. Enhanced levels of VEGF and other angiogenic factors from concentrated platelets stimulate the formation of new blood vessels, improving blood supply to the damaged tissue and supporting healing [45-46].

In a prospective single-arm interventional study by Jain et al., 20 patients (12 men, 8 women, mean age: 35 years) with discogenic pain received PRP injections at single or multiple disc levels. MRI findings of each patient included high-intensity zone, decreased signal intensity in intervertebral discs on T2 imaging, intervertebral disc protrusion, loss of intervertebral disc height, and end plate changes. Platelet counts of patients and PRP samples were measured. Researchers found that at three-month and six-month follow-ups, the improvement in pain and disability scores positively correlated with platelet concentrations in the PRP sample [47].

A retrospective study by Lutz et al. included 37 patients with chronic discogenic low back pain who received intradiscal injections of higher concentration (>10×) PRP. Outcomes were compared to a historical cohort of 29 patients who received intradiscal injections of <5x PRP. Pain, functionality, and satisfaction were significantly improved in patients who received a high concentration of PRP. The authors concluded that “clinical outcomes can be optimized by using PRP preparations that contain a higher concentration of platelets” [48]. A summary of the included studies is provided in Table 2.

**Table 2:** Summary of the included studies Ro: X-ray; MRI: magnetic resonance imaging; PRP: platelet-rich plasma

First author	Year	n	Intervention	Follow-up	Results
Akeda et al. [33]	2017	14	Intradiscal PRP injection	1, 2, 6 months	More than 50% reduction of low back pain was observed in 71% of patients within 1 month. Seventy-nine percent of patients showed a more than 50% reduction in physical disability scores within 1 month. No significant Ro/MRI changes.
Kirchner et al. [34]	2021	32	Intradiscal and epidural PRP injection	1, 3, 6 months	Pain reduction of more than 30% was observed in 78% of patients in 1 month after treatment and in 87% of patients at 6 months after treatment.
Akeda et al. [35]	2022	11	Intradiscal PRP injection	5.9 years	In 91% of patients, more than a 30% improvement in pain intensity and physical disability, were identified. A 14% decrease in disc height compared to baseline was recorded.
Levi et al. [36]	2016	22	Intradiscal PRP injection	1, 2, 6 months	A 50% improvement in the pain intensity and a 30% decrease in the Oswestry Disability Index was observed in 14% of patients at 1 month, in 32% of patients at 2 months, and in 47% of patients at 6 months after injection.
Akeda et al. 37]	2022	16	Intradiscal PRP injection versus corticosteroid injection	1, 2, 6 months	PRP group had a significantly improved disability score at 26 weeks and walking ability scores at 1 and 2 months. No significant differences in Ro/MRI.
Cheng et al. [38]	2019	19	Intradiscal PRP injection	5-9 years	Significant improvements in pain and function (p < 0.001). Fifty-eight percent patient satisfaction.
Zhang et al. [39]	2022	31	Intradiscal PRP injection	1-48 weeks	PRP injection was associated with a significant improvement in pain intensity and lumbar function.
Zielinski et al. [40]	2022	26	Intradiscal PRP injection versus saline	2 months	No significant superiority of PRP injections in terms of pain relief or functionality improvement.
Akeda et al. [41]	2023	15	Intradiscal PRP injection versus corticosteroid injection	1 year	PRP group had significantly improved lower back pain and disability. No significant differences in Ro/MRI.
Jayasoorya et al. [42]	2024	64	Epidural PRP injections versus corticosteroid injection	3 months	PRP group had a significantly decreased pain intensity.
Navani et al. [43]	2024	40	Intradiscal PRP injection versus bone marrow concentrate vs saline	1, 3, 6, 12 months	Both PRP and bone marrow concentrate were associated with statistically significant improvement in pain and function in comparison to placebo.
Comella et al. [44]	2017	15	Intradiscal PRP injection versus stromal vascular fraction	1 year	Pain intensity, flexion, and quality of life were significantly improved in the PRP group.
Jain et al. [47]	2020	20	Intradiscal PRP injection	1, 3, 6 months	The improvement in pain and disability scores positively correlated with platelet concentrations in the PRP sample.
Lutz et al. [48]	2022	37	Intradiscal PRP injections: Higher-concentration (>10×) versus lower concentration (<5×)	Mean: 18-months	Pain, functionality, and satisfaction were significantly improved in patients who received a high concentration of PRP.

## Conclusions

PRP therapy represents a promising avenue for tissue healing and regeneration across various medical specialties. It has been found to promote tissue regeneration and modulate inflammatory response in degenerated discs. PRP can be administered mostly intradiscally but also epidurally. The benefits of PRP use include pain reduction, improvement of functionality, and low risk of adverse events. The effect of intradiscal PRP injections is similar to steroid injections. A higher concentration of platelets is associated with enhanced clinical outcomes. While the evidence supporting its efficacy is encouraging, further research is needed to elucidate its mechanisms of action, optimize treatment protocols, and expand its clinical applications.
